# 

**Published:** 1904-02-20

**Authors:** 


### Publications.

Just Published. 8vo. with Photographic reproductions, 3/- net.

**THE PHYSIOGNOMY OF MENTAL DISEASES AND DEGENERACY.** By James Shaw, M.D., formerly Med. Supt. and Co-Licensee Haydock Lodge Asylum. Reprinted, with alterations and additions, from various articles in the "Medical Annual."

Just Published. Crown 8vo. 1/6 net.

**A POCKET BOOK OF CLINICAL METHODS.** By Chas. H. Melland, M.D.Lond., M.R.C.P., Phys. Ancoats Hosp.

THIRD EDITION. 12th Thousand. Small 8vo. Illustrated with 200 Original Drawings, 2/6. Lantern Slides of the Illustrations, 1/- and 1/6 each.

**"FIRST AID" TO THE NJURED AND SICK:** An Advanced Ambulance Handbook. By F. J. Warwick, B.A., M.B., and A. C. Tunstall, M.D., F.R.C.S.

THIRD EDITION. 2/- each, or 27/6 the Set of 16 Sheets, or mounted on linen, 45/- With nickel head.

**(Adopted by the War Office, Admiralty, and London School Board.)**

**LARGE SHEET "FIRST AID" DIAGRAMS:** Being Enlargements of the Illustrations in the above work on sheets 26 in. by 40 in., suitable for lectures and classes.

**Bristol: JOHN WRIGHT & CO.**

**MEDICAL ACCOUNTS.**

Now is the time to arrange for this troublesome business, and

**WRIGHT'S THREE-BOOK SYSTEM,**
**WRIGHT'S SINGLE-BOOK SYSTEM,**
(11th Edition)
**WRIGHT'S OBSTETRIC RECORDS,**
**WRIGHT'S VISITING LISTS, 1904,**
**WRIGHT'S CHARTS of all kinds,**
(Nearly 400,000 of which have been sold)
**WRIGHT'S CHART HOLDERS,**
(In use all over the world)
**WRIGHT'S CERTIFICATE BOOKS,**
**PROFESSIONAL ACCOUNT FORMS,**
**LETTER HEADINGS, and**
**DISPENSING LABELS.**

Are most of them better than the ordinary run of these things, and their use saves time and trouble.

PLEASE SEND FOR FREE SAMPLES.

**London: SIMPKIN & CO., Ltd.**

FOURTH EDITION. Plates contain 93 Coloured and Stereoscopic Figures, 209 Illustrations in the Text, 17/6 net, including Stereoscope.

By P. WATSON WILLIAMS, M.D.(Lond.),
Physician in Charge of Throat Department,
Bristol Royal Infirmary, &c., &c.

**DISEASES OF THE NOSE, PHARYNX, AND LARYNX.**

WITH A SECTION ON THE EXAMINATION OF

**THE EAR.**

**Press Notices.**—"We know of no better practical guide."—*Practitioner.* "Certain to receive the recommendation of all teachers."—*Brit. Med. Journ.* "We can strongly recommend the book."—*Lancet.* "Excellent......The features of the work—completeness, clear description, and practical utility—are absolutely maintained in the new edition.—(Transn.)." *Internat. Centr. f. Laryngologie.*

Bristol: J. WRIGHT & CO. London: SIMPKIN & CO.

Crown 8vo. Illustrated. Price **2s. 6d.** net.

**ANÆSTHETICS.** By J. Blumfeld, M.D.Cantab., Senior Anæsthetist to St. George's Hospital; Anæsthetist to the Grosvenor Hospital for Women and Children, London, and to the National Dental and Metropolitan Hospitals.

*Lancet.*—"....In writing this handbook the author has been quite successful." *Australasian Medical Gazette.*—"We can strongly commend this book." *Medical Chronicle.*—"The book is one which we can heartily recommend to senior students and practitioners."

London: Baillière, Tindall & Cox, 8 Henrietta St., Covent Garden.

Second Edition. Crown 8vo., price **3/6** net.

With original "Plea—1900—for the State Registration of Nurses."

**MOCK NURSES OF THE LATEST FASHION.**

By FREDERICK J. GANT, F.R.C.S.,
Consulting Surgeon to the Royal Free Hospital.

BAILLIÈRE, TINDALL & COX,
Henrietta Street, Covent Garden, London.

**Hoblyn's Dictionary of TERMS USED IN MEDICINE and the Collateral Sciences.**

13th Edition, Revised by J. A. P. Price. **10/6.**

"It has a rare merit of supplying in almost every case what you have a right to expect in consulting it."—*Glasgow Medical Journal.*
"As a handy reference volume for the physician, surgeon and pharmacist it will prove invaluable."—*Pharmaceutical Journal.*

**THE APOTHECARIES' HALL MANUAL FOR Students preparing for the Dispenser's Certificate. By M. THOMSON, 2 - net.**

WHITTAKER & CO., White Hart St., Paternoster Sq. London, E.C.

### NOTICE TO CORRESPONDENTS.

All MSS., Letters, Books for Review, and other matters intended for the Editor should be addressed The Editor, "The Hospital," The Hospital Buildings, 28 & 29 Southampton Street, Strand, W.C.

The Editor cannot undertake to return rejected MS., even when accompanied by stamped directed envelope.

**Notice to Advertisers and Subscribers.**

All Advertisements, Orders for Copies of The Hospital, and Business Communications should be addressed to **THE MANAGER (Not to the Editor),** The Hospital, 28 & 29 Southampton Street, Strand, London, W.C.

The Cost of Subscription, post free, is as follows:—Per week, 3½d.; per quarter, 3s. 9d.; per half-year, 7s. 6d.; per annum, 15s. Foreign and Colonial:—Per annum, 19s. 6d., payable, in all cases, in advance.

**NOTICE.—Vol. XXXIV. of "The Hospital" (April 1903 to September 1903), in handsome cloth binding, is now on sale at the office of this Journal, price 10s. 6d. Cases for binding the Numbers contained in this Volume 2s. Index to Vol. XXXIV., 2½d., post free.**

**The Monthly part for February is now ready. Price 1s., by post, 1s. 3d.**

### BOOKS RECEIVED.

Baillière, Tindall and Cox.

"Midwifery for Midwives." By W. D. Wiggins.

"Drugs: their Production, Preparation, and Properties." By H. Wippell Gadd.

J. Bale, Son, and Danielson.

"Vaginal Tumours." By W. Roger Williams.

Hickson, Ward and Co.

"Report of the Select Committee on Ventilation."

University Tutorial Press.

"London Matriculation Directory." No. XXXVI., Jan., 1904.

### Annual Meeting.

THE CANCER HOSPITAL (FREE), FULHAM ROAD, S.W.

The Annual General Meeting of the Governors, Lord Ludlow, President, in the Chair, will be held in the Board Room on Wednesday, the 24th inst., at 4 p.m. precisely. FRED W. HOWELL, Secretary.

### The Student of Medicine.

APPOINTMENTS.

**S. E. Atkins**, L.R.C.S.I., L.S.A., Certifying Factory Surgeon for the Hatherleigh District, Devonshire. **C. B. Blackburn**, M.D., Ch.M.Syd., Assistant Physician to Royal Prince Alfred Hospital, New South Wales. **George P. Bletchley**, M.B.Lond., M.R.C.S.Eng., L.R.C.P.Lond., Medical Officer and Public Vaccinator for Third Division of the Stroud Union, and Certifying Factory Surgeon for the Nailsworth District. **G. Candler**, M.R.C.S., L.R.C.P.Lond., District Medical Officer of the Holsworthy Union. **F. A. Davies**, M.B., District Medical Officer of the Hexham Union. **E. W. Fairfax**, M.B., Ch.M.Syd., Assistant Physician to Royal Prince Alfred Hospital, New South Wales. **A. F. Goldsmith**, M.R.C.S., L.R.C.P.Lond., District Medical Officer of the Bedford Union. **Frank Heasman**, M.R.C.S.Eng., L.R.C.P.Lond., Assistant Physician to the Royal Boscombe and West Hants Hospital. **L. Capper Johnson**, M.B.Lond., M.B., Ch.B.Vict., M.R.C.S., L.R.C.P., Medical Officer to the Central Home for Children, Leeds Union. **Ernest Le Cronier Lancaster**, B.A., M.B., B.Ch.Oxon., M.R.C.S.Eng., Physician to the Swansea General and Eye Hospital. **A. H. Macintosh**, M.B., Ch.M.Syd., Medical Superintendent to Royal Prince Alfred Hospital, New South Wales. **F. Victor Milward**, M.B., B.C.Cantab., F.R.C.S.Eng., Medical Referee under the Workmen's Compensation Act for Birmingham and for Bromsgrove, Redditch, and Solihull. **A. W. Nuthall**, F.R.C.S., Surgeon to Out-patients to the Birmingham and Midland Free Hospital for Sick Children. **C. A. Palmer**, M.R.C.S., L.R.C.P.Lond., Medical Officer of Health for the Newbold and Dunstan Urban District. **F. J. Pearson**, M.R.C.S., L.R.C.P., District Medical Officer of the Gainsborough Union. **L. J. Picton**, M.B., B.Ch.Oxon., District Medical Officer of the Congleton Union. **J. Pringle**, M.D., B.Ch.Dub., Certifying Factory Surgeon for the Manchester S.W. District. **J. F. Watson**, M.B., M.Ch.Melb., Assistant Medical Superintendent to Hospital for the Insane, Toowoomba, Queensland. **H. M. Wilson**, M.B., B.C.Cantab., Assistant Medical Officer to Wellington Hospital, New Zealand. **J. Clark Wilson**, M.D., C.M.Edin., D.P.H.Camb., Pathologist to Friedenheim Hospital, Upper Avenue Road, N.W.

**"The Hospital" Institutional Library.**

"Hospitals and Asylums of the World." 4 Vols., with a Portfolio of Plans, complete £12 12s.

"Burdett's Hospitals and Charities, 1903." 5s. net.

"Burdett's Official Nursing Directory, 1903." 8s. net.

"Cottage Hospitals, General, Fever, and Convalescent." 10s. 6d.

"Uniform System of Accounts for Hospitals and Public Institutions." New Edition. 4s. net.

"Hospital Expenditure: The Commissariat." 2s. 6d.

"A Legal Handbook for the Use of Hospital Authorities." 2s. 6d.

"The Cottage Hospital Case Book or Register of Patients." 10s. and 12s. 6d.

All these are published by the Scientific Press, Ltd., and may be obtained through any bookseller, or direct from the publishers, 28 and 29 Southampton Street, Strand, London, W.C.

How to Become a Nurse.

### THE NURSING PROFESSION:

HOW AND WHERE TO TRAIN.

By SIR HENRY BURDETT, K.C.B.

Is a reliable and up-to-date Guide to Training for the calling of a Nurse. In its pages would-be Nurses will find all the information they require.

Price 2/-, post free 2/4.

Of all Booksellers or of THE SCIENTIFIC PRESS, LTD., 28 & 29 Southampton Street, Strand, London, W.C.

### To Nurses.

### A NEW CASE BOOK FOR PRIVATE NURSING.

**Arranged for Day and Night Nurse's Report, with rulings in accordance with what has been found in practice to be the most suitable and approved System for Pay Hospitals, Nursing Homes, Private Nurses, &c.**

DAY NURSE'S REPORT.

*Date............*

| Time | Nourishment | Quantity | Urine | Stimulants, Medicines, and Remarks |
|---|---|---|---|---|
| | | | | |

| Total Nourishment | Bowels | Summary |
|---|---|---|
| | | |

(Facsimile of ruling.)

**Price 3d. net.** Post free 4½d.

**The Size of an Exercise Book (7½ in. by 9½ in.).** *Obtainable of all Booksellers.*

**Price 3d. net** Post free 4½d.

*Special Quotations for a quantity can be procured from the Publishers.*

THE SCIENTIFIC PRESS, LTD., 28 & 29 Southampton Street, Strand, London, W.C.

### THE BURDETT SERIES OF POPULAR TEXT-BOOKS on NURSING.

Small Crown 8vo. Bound in cloth boards. Price **1/-** each post free; or the Series complete **10/-** post free.

**Each Volume contains about 100 pages written by a competent authority on the subject treated.**

**"Valuable little handbooks."**
*Nursing Notes.*

Price **1/-** each Post Free.

**"Can be strongly recommended to nurses."**
*Treatment.*

No. 1.—**PRACTICAL HINTS ON DISTRICT NURSING.** By AMY HUGHES.

No. 2.—**HOSPITAL AND PRIVATE NURSING.** By S. E. ORME.

No. 3.—**THE MIDWIVES' POCKET-BOOK. With Prefatory Chapter on the Midwives' Bill.** By HONNOR MORTEN, Author of "The Nurse's Dictionary," &c., &c.

No. 4.—**FEVERS AND INFECTIOUS DISEASES:** their Nursing and Practical Management. By WILLIAM HARDING, M.D.Ed., M.R.C.P.Lond., Author of "Mental Nursing," &c.

No. 5.—**NOTES ON PHARMACY AND DISPENSING FOR NURSES.** By C. J. S. THOMPSON.

No. 7.—**THE CARE OF CONSUMPTIVES.** By W. H. DAW, M.R.C.S., L.R.C.P.Lond.

No. 8.—**HOME NURSING OF SICK CHILDREN.** By J. D. E. MORTIMER, M.B., F.R.C.S., L.S.A.

No. 9.—**MENTAL NURSING.** By WILLIAM HARDING, M.D.Ed., M.R.C.P.Lond.

No. 10.—**SICK NURSING AT HOME.** By Miss L. G. MOBERLY.

No. 11.—**GYNÆCOLOGICAL NURSING.** By G. A. HAWKINS-AMBLER, F.R.C.S., Author of "The Commonwealth of the Body."

No. 12.—**SYLLABUS OF LECTURES TO NURSES.** By ANDREW DAVIDSON, M.D.

*These books are obtainable of all Booksellers and are published by*
THE SCIENTIFIC PRESS, LTD., 28 and 29 Southampton Street, Strand, London, W.C.,
*who will send their latest Catalogue of Nursing Manuals, post free, to any Nurse on application.*

### WORKS FOR NURSES.

**LESSONS ON MASSAGE.** By Mrs. MARGARET D. PALMER, Manager of the Massage Department and Instructor of Massage to the Nursing Staff of the London Hospital. Second Edition enlarged and revised; with 118 Illustrations. Price 7/6 net.

**A MANUAL FOR STUDENTS OF MASSAGE.** By Miss M. A. ELLISON, L.O.S. Second Edition, edited by Miss G. MANLEY, pp. xii + 126. With 50 original Illustrations. Price 3/6 net.

**A MANUAL FOR HOME NURSING.** By BERNARD MYERS, M.D., M.R.C.S., Surgeon and Lecturer to St. John's Ambulance Association. 2/6 net.

**NOTES ON HOME NURSING.** By Miss D. M. GOLDIE, Lecturer on Nursing, Hygiene, &c., to the County Councils of London, Surrey, &c. 1/6 net.

**NUTRITION OF THE INFANT.** By RALPH VINCENT, M.D., M.R.C.P., Physician to the Infants' Hospital.

**THE FEEDING AND HYGIENE OF INFANTS AND CHILDREN.** By JOHN McCAW, M.D., M.R.C.P., Physician to the Belfast Hospital for Children. *Just Published.* Price 2/6.

**MIDWIFERY FOR MIDWIVES.** Designed to cover the requirements of the Examining Board authorised by the Midwives Act. With Appendix on Examinations. By W. DENISON WIGGINS, M.R.C.S., L.R.C.P., &c. *Just out.* 3/6 net.

**DISEASES OF THE HAIR, INCLUDING GREYNESS, BALDNESS, &c., AND THEIR TREATMENT.** By DAVID WALSH, M.D., Western Hospital for Diseases of the Skin. Price 2/6 net.

**MENSTRUATION AND ITS DISORDERS.** By ARTHUR E. GILES, M.D., B.Sc., F.R.C.S., Surgeon Chelsea Hospital for Women. Price 2/6 net.

**MOVABLE ATLAS OF THE ANATOMY AND PHYSIOLOGY OF THE FEMALE GENERATIVE ORGANS AND OF PREGNANCY.** Second Edition. With Text. By ARTHUR E. GILES, M.D., F.R.C.S. Price 3/- net.

**MOVABLE ATLAS OF THE ANATOMY AND PHYSIOLOGY OF THE CHILD.** With Explanatory Text. By D'ARCY POWER, M.A.Oxon, M.B., F.R.C.S. Price 3/- net.

London: BAILLIÈRE, TINDALL & COX, 8 Henrietta Street, Covent Garden.

